# A large cross-sectional study on the prevalence and predictors of donor and donation images in patients after heart transplantation

**DOI:** 10.1038/s41598-025-07317-7

**Published:** 2025-07-01

**Authors:** Nora M. Laskowski, Georg Halbeisen, Leoni Wahlers, Katharina Tigges-Limmer, Georgios Paslakis

**Affiliations:** 1 Medical Faculty, University Clinic for Psychosomatic Medicine and Psychotherapy, Ruhr-University Bochum, Campus East- Westphalia, Virchowstr. 65, 32312 Luebbecke, Germany; 2 Clinic for Thoracic and Cardiovascular Surgery, Herz- und Diabeteszentrum NRWRuhr-University Bochum, Bad Oeynhausen, Germany

**Keywords:** DDI, Donor image, Donor fantasies, Organ fantasies, Heart transplantation, Psychocardiology, Psychology, Cardiology

## Abstract

Recovery and clinical outcomes following organ transplantation may be negatively influenced by psychological distress. Limited reports from recipients indicate that thoughts and feelings related to the donor/transplanted organ – referred to as donor and donation images (DDI) – may be a source of such distress. The term DDI encompasses all thoughts and emotions that organ recipients associate with the donor or the donated organ. However, empirical knowledge of DDI remains limited. This quantitative survey involving 407 participants represents the first and largest study to quantitatively examine DDI in patients after heart transplantation (HTX). The results revealed a very high prevalence of DDI (91%), with occurrences reported intermittently and often clustered around the time of transplantation, both before and after HTX. Psychological distress predicted the occurrence of DDI before and after HTX and almost all emotions experienced and reported pre-HTX were associated with higher odds of pre-HTX DDI, suggesting it may be a concurrent phenomenon of overall emotional activation. Due to the involvement of emotions associated with uncertainty and low personal control, along with high situational control, DDI may be a part of coping. Some reported avoiding DDI suggesting that, sometimes, they might also represent a stressor. Future studies should further investigate the effects of DDI, including its impact on transplant outcome.

**Supplementary Information:**

The online version contains supplementary material available at 10.1038/s41598-025-07317-7.

## Introduction

The impact of heart transplantation (HTX), the state-of-the-art treatment for heart failure, severe injury, or irreversible disease, extends beyond merely replacing a malfunctioning body part. Rather, heart transplantation is a major intervention with profound consequences, including significant psychological and physical stress. Physical stress may involve rejection reactions, which, in turn, are associated with high mortality^1,2^.

In terms of psychological distress, studies indicate that symptoms of anxiety, particularly fear of organ rejection, re-operation, and death, along with depression, are most commonly reported among recipients^2–4^. For instance, approximately 10% of patients following HTX exhibits symptoms of post-traumatic stress disorder (PTSD), such as intrusions, flashbacks, flooding emotions, nightmares, high inner tension, and avoidance^5^. PTSD symptoms emerge due to high distress associated with transplantation, such as intensive care treatment, accompanied by a consistently perceived acute threat of death^6^.

Previous studies have shown that critical care experiences can lead to significant psychological distress, including symptoms of anxiety, depression, and PTSD, both in general intensive care unit populations^7,8^ and in patients with cardiovascular diseases^9^. However, HTX involves additional psychological challenges^10^. In contrast to other organs, the heart holds deep symbolic and emotional meaning -it is often regarded as the seat of emotions, identity, and personality^11–13^, which may intensify psychological distress. Consequently, HTX recipients not only face the physical challenges of a life-threatening intervention but must also cope with the psychological task of integrating a heart from a deceased donor into their own body^14^. This process can evoke intense emotional reactions, including guilt, indebtedness, fear of personal or emotional changes, but also deep gratitude^12^. The psychological, HTX-related distress is further linked to a significantly lower quality of life^15^ and can have serious consequences for the recovery progress, including negatively impacting transplant success^3,16–18^.

Although the factors determining the psychological and physical integration of a transplanted organ are still not fully identified, qualitative patient reports indicate that thoughts and upcoming feelings related to the organ donor and/or the transplanted organ itself may constitute a potential source of psychological distress^2,12,18,19^. Some authors have referred to this as “fantasies about the donor” (e.g.,^20^), or “fantasies about the organ” (e.g.,^21^). However, Laskowski et al. recently introduced the more neutral and descriptive term “Donor and Donation Images” (DDI) since DDI involves actual thoughts rather than mere fantasies^12^. The term DDI was introduced to describe all thoughts and emotions that organ recipients associate with the donor and/or the donated organ, avoiding the potentially pathologizing connotation of the term “fantasy". As defined in Laskowski et al., DDI may stem from internally constructed imagery but also from actual information (if available in the respective country) or emotionally charged experiences during the transplantation process^12^. Given this complexity, the concept captures a psychological phenomenon that is not merely in the mind but often anchored in elements of lived experience.

A previously conducted literature review in 2023 highlighted that limited knowledge on DDI^12^. For instance, crucial aspects such as the prevalence of DDI, the level of psychological distress (due to emotional activation) or subjective relief (in terms of an emotional coping), or the extent to which attributions to the donor are connected to perceived behavioral and experiential changes remain understudied. Existing findings are sparse and primarily originate from qualitative investigations (see e.g.,^11,17,21,22^).

A relevant questionnaire in the context of post-transplant adjustment is the Transplant Effects Questionnaire (TxEQ;^23^), developed to assess emotional and behavioral responses after transplantation across five key domains: worry about the transplant, guilt regarding the donor, disclosure, perceived responsibility, and medication adherence. However, the TxEQ does not specifically capture aspects of donor- and/or organ-related thoughts and/or symbolic meanings associated with the transplanted organ. As no standardized measure currently exists to assess DDI, we have created a specific set of questions tailored to assess the multidimensional nature of this construct. This highlights both a methodological gap and a substantial need for systematic research to explore the prevalence, timing, intensity, and psychological implications of DDI across the transplantation process^12^.

Given the partial interrelation between psychological and physiological organ rejection reactions^17^, gaining a deeper understanding of DDI may prove essential for HTX patient care, recovery, and transplant success. The present study is the first to systematically explore this line of research. Specifically, the study aimed to establish a data basis for the development of a taxonomy of DDI, serving a basis for further theory building. Additionally, the study tested the following hypotheses: (1) DDI increase post-HTX compared to pre-HTX, and (2) psychological distress is correlated with the occurrence of DDI.

## Methods

### Survey design

Due to the absence of existing questionnaires for assessing DDI, this cross-sectional survey employed a self-developed questionnaire focused on transplantation experience. Questions were formulated based on descriptions of recipients’ HTX experiences from previous interview studies (e.g.,^11,17,21,22^). Additionally, the questionnaire included novel aspects regarding DDI and its associated burden or coping mechanisms. The questions relevant for this study covered the following domains:

1. *Recipient information* (11 items, results are presented in Table 1): Participants provided basic sociodemographic information including age (in years), gender (man, woman, diverse, queer, prefer not to say), and their romantic attraction (with multiple responses possible: women, men, other genders). Language proficiency was assessed by asking about their level of German (native speaker, fluent, basic knowledge). Migration background was assessed with the question: “Do you or at least one of your parents have a country of birth outside of Germany?” (yes/no). Educational background was captured through years of schooling (< 12/≥12). Further items addressed marital status (single, married/civil partnership, widowed, divorced), current relationship status (yes/no), and household composition (single-person, two-person, multi-person household). Finally, participants were asked to report their weight and height, which were used to calculate the Body Mass Index (BMI).
2. *Transplantation process* (5 items, results are presented in Table 1 and Sect. 3.2): Participants were asked to indicate the month and year of their HTX to calculate their age at the time of the procedure. The waiting time prior to transplantation was reported in months and the length of the inpatient hospital stay following the HTX was reported in days, both as a free-text response. Participants indicated whether any complications occurred during or after the transplantation (yes/no). Participants also rated how physically and psychologically burdensome the hospital stay was, using two separate 11-point Likert-type scales ranging from 0 = not burdensome at all to 10 = extremely burdensome. Finally, participants were asked whether they had been diagnosed with a stress-related or anxiety disorder following the transplantation (yes/no).
3. *Thoughts related to the surgical procedure* (3 items, results are presented in Sect. 3.3): To assess the cognitive engagement with the transplantation process, participants were asked to retrospectively report how often they thought about the surgical procedure itself, both before and after their HTX. Response options included: at least once a day, at least once a week, at least once a month, less frequently, only once, never. In addition, participants indicated the typical duration of such thoughts when they occurred, selecting from the following categories: a few seconds, a few minutes, several minutes, hours.
4. *DDI* (12 items): Participants were asked whether they had ever mentally engaged with the person who donated the organ, both before and after HTX (yes/no). Those who indicated having DDI were asked to rate (a) the frequency of these thoughts both before and after the HTX, with the following options: only once, recurring in phases, permanent, never (results are presented in Sect. 3.4), (b) the time of the occurrence of DDI, before and after HTX, choosing from: immediately before/after transplantation, days/weeks/months/years before or after transplantation, never (results are presented in Sect. 3.5), (c) the frequency of DDI, both before and after HTX, using a 6-point scale (at least once a day, once a week, once a month, less frequently, only once, never), and (d) the time they typically spent with DDI when they occurred, choosing from: a few seconds, a few minutes, several minutes, hours, or such thoughts never occurred (results are presented in Sect. 3.6). Finally, all participants were asked whether they had ever actively tried to avoid thinking about the donor -both before and after transplantation- and, if so, whether these avoidance attempts had been successful (yes, no/the thoughts persisted, I did not try to avoid them, or I cannot remember; results are presented in Sect. 3.7).
5. *Emotions related to the transplantation and donor* (33 items, results are presented in Table 2 and Sect. 3.8): To assess emotional experiences related to the transplantation process and the donor, participants were asked to rate their agreement with a series of emotion-focused statements referring to three time points: (a) in anticipation of the transplantation, (b) after the transplantation, and (c) when thinking about the donor. At each time point, participants indicated the extent to which they experienced the following emotions: hope, joy, fear, shame, guilt, sadness, anger, rage, anxiety, disgust, and gratitude. Responses were recorded using a 4-point Likert-type scale: does not apply at all, rather does not apply, rather applies, fully applies.
6. *Personal beliefs and perceptions* (13 items, results are presented in Table 3 and Sect. 3.9): To explore participants’ beliefs and symbolic representations related to the heart, the body, and the concept of the soul, a set of 13 statements was presented (see Table 3). Participants were asked to indicate their level of agreement on a 4-point Likert scale: strongly disagree, somewhat disagree, somewhat agree, strongly agree.

### Participants and ethics

The participants in this study did not receive any compensation for their participation. All participants gave informed written consent and declared their full legal capacity. Anonymity was assured to minimize potential response bias and expectation effects. The study included all individuals from the existing pool of patients who had undergone HTX at the Clinic for Thoracic and Cardiovascular Surgery (“Herz- und Diabeteszentrum NRW”) at any time, and who were registered as alive (*N* = 1023). Only adults (≥ 18 years) with sufficient knowledge of German were eligible for inclusion.

All relevant ethic statements and guidelines were adhered to within the study, which had been reviewed and approved by the Ethics Committee of the Medical Faculty of the Ruhr-University Bochum (AZ 2022 − 959). The study has been performed in accordance with the Declaration of Helsinki and was prospectively registered with AsPredicted under application #112,370.

The survey was distributed from January 17th to January 24th, 2023, and was accompanied by a prepaid return envelope.

### Statistical analysis

Descriptive results are presented as frequencies and percentages for categorical variables, and as the mean and standard deviation (*SD*), as well as the median plus range for continuous variables. Since no standardized instrument for assessing DDI currently exists, a self-constructed questionnaire was created specifically for this study. As a result, no psychometric reliability analysis was performed.

Predictors of DDI pre- and post-HTX were determined through binary logistic regressions, with the dependent variable being DDI pre-HTX (“yes” [= 2]/"no” [= 1]) and DDI post-HTX (“yes” [= 2]/"no” [= 1]), respectively. The formation of the dependent variable was based on the question “How often did DDI occur before/after HTX?” with “never” coded as “no (=1)” and all other responses coded as “yes” (= 2). For the regressions, the presence of emotions and data for personal beliefs and perceptions were dichotomized, with two responses each indicating agreement and disagreement (“strongly agree”/“agree”=“yes” [= 2], “disagree”/"strongly disagree”=“no” [= 1]). Metric variables were entered as such in the regressions.

Statistical analyses were performed using SPSS Statistics version 28^24^ and R version 4.2.1^25^. All available data sets were included (missing data < 4.4%, missing completely at random). The significance level for all analyses was set at *p* ≤ .05. Data sets are available upon request from the corresponding author.

## Results

### Sociodemographic variables

A total of 416 individuals completed the survey questionnaire (response rate 40.7%). One underage person was excluded, along with an additional eight individuals who completed less than 50% of the DDI-relevant questions. Consequently, 407 datasets were included in the analysis. Table 1 provides sociodemographic information and details related to the transplantation process.

### Burden of the heart transplantation

Participants indicated on a scale from 0 (“no burden”) to 10 (“highest possible burden”) the burden of their inpatient stay following HTX. The psychological burden (mean = 5.19, *SD* = 3.09, 9 missing responses) was reported as high as the physical burden (mean = 5.18, *SD* = 3.03, 8 missing responses). Furthermore, 14.6% stated that they had been diagnosed with and anxiety disorder or depression post-HTX (*n* = 58, 9 missing responses).

### Surgical procedure

First, the frequency of patients’ preoccupation with the surgical procedure was assessed before and after HTX. Before the surgery, a total of 7.5% did not think about the upcoming transplantation at any time (“never”, *n* = 27, 46 missing responses), and 2.8% only thought about it “once” (*n* = 10). The majority of participants thought about the HTX on a “daily” basis (44.9%, *n* = 162), followed by “at least once a week” (20.8%, *n* = 75), “at least once a month” (5.3%, *n* = 19), and even “less frequently” (18.8%, *n =* 68).

After the surgery, 22 participants were not mentally preoccupied with the surgical procedure at any time (“never”, 5.5%, 6 missing responses), and 1.7% only thought about it “once” (*n* = 7). Again, the highest percentage of recipients thought about the HTX “daily” (33.7%, *n* = 135), followed by “at least once a week” (23.2%, *n* = 93), “at least once a month” (9.7%, *n* = 39), and even “less frequently” (26.2%, *n* = 105). Eight recipients denied having any thoughts regarding the surgery (both before and after) whatsoever (2.2%, 47 missing responses).

Nine recipients stated that they had thought about the surgery before but not after it had taken place (2.5%), and conversely, 19 recipients had no relevant thoughts about the surgery before HTX but did develop them after HTX (5.3%).

To the extent that the thoughts occurred, they mostly lasted for “a few” (50.7%, *n* = 190, 32 missing responses) or “several” (29.1%, *n* = 109) minutes. Fewer reported thoughts lasting shorter (“a few seconds”, 16.8%, *n* = 63) or longer (“hours”, 3.5%, *n* = 13).

### Prevalence of donor and donation images

The occurrence of DDI was assessed before and after HTX. Among all recipients, 59.8% reported DDI prior to the HTX (*n* = 202, 69 missing responses), which increased to 91.0% post-HTX (*n* = 364, 7 missing responses).

Among the 336 participants who provided valid responses both before and after HTX, 198 (58.9%) reported experiencing DDI at both time points. Only two participants who reported DDI pre-HTX did not report DDI post-HTX (0.6%). Twenty-seven recipients reported no DDI either pre- or post-HTX (8.0%) A total of 109 recipients had no DDI pre-HTX but reported the occurrence of DDI afterwards (32.4%). In summary, the hypothesis that DDI increase post-HTX may be confirmed.

**Fig. 1:**
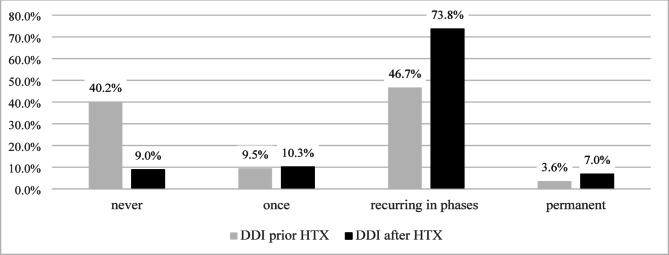
Prevalence of donor and donation images before/after heart transplantation. *DDI = Donor and donation images; HTX = heart transplantation.*

Most respondents indicated that DDI occur “in phases”. For 29.5% of respondents, DDI persist to the present (*n* = 75). Figure 1 displays the frequency of DDI before and after HTX, showing a clear increase in DDI after HTX.

### Time of occurrence of donor and donation images

**Fig. 2:**
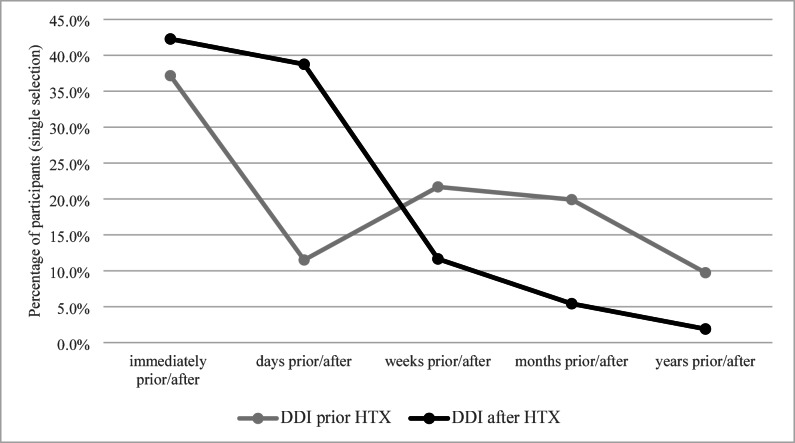
Time of occurrence of donor and donation images before/after heart transplantation. Participants were asked to select only one time frame. Percentages reflect mutually exclusive responses and therefore sum up to 100% per time frame. *DDI = Donor and donation images; HTX = heart transplantation.*

To identify the most salient phase of DDI occurrence, only participants who reported experiencing DDI were asked to select a single time frame during which DDI occurred most frequently -both before and after HTX. As shown in Fig. 2, prior to HTX, 27.1% of participants reported experiencing DDI immediately before the procedure, followed by 24.7% in the weeks before, 25.3% in the months before, 12.0% in the years before, and 10.8% in the days before.

After HTX, DDI were most frequently reported to occur immediately after (42.7%) or in the days after (38.7%). Fewer participants indicated the weeks after (12.1%), months after (5.1%), or years after (1.4%) as the most prominent phase. These findings suggest that DDI predominantly cluster around the perioperative phase, with the highest occurrence shortly before and after transplantation.

### Intensity of donor and donation images

To identify the most salient phase of DDI occurrence, only participants who reported experiencing DDI were asked to indicate how frequently these thoughts occurred. Among recipients in whom DDI occurred pre-HTX (*n* = 202), a total of 164 provided responses regarding the intensity of these experiences. DDI occurred “at least once a day” in 33.5% (*n* = 55) and “at least once a week” in 22.6% (*n* = 37). Slightly fewer reported DDI occurring “at least once a month” (11.0%, *n* = 18), “less frequently” (23.8%, *n* = 39), or only “once” (9.1%, *n* = 15).

Among recipients in whom DDI occurred post-HTX (*n* = 364), a total of 352 provided responses regarding the intensity of these experiences. Post-HTX, DDI mostly occurred “less frequently” than once a month (31.0%, *n* = 109), followed by “at least once a day” (30.4%, *n* = 107) or “at least once a week” (26.4%, *n* = 93). DDI occurred “at least once a month” in 8.2% (*n* = 29), and only “once” in 4.0% (*n* = 14).

The duration of singular thoughts was reported by almost half of recipients as being “a few minutes” long (50.0%, *n* = 184, 39 missing responses), followed by “several minutes” (28.8%, *n* = 106). Less frequently, DDI were reported to last a “few seconds” (18.8%, *n* = 69) or “hours” (2.4%, *n* = 9).

### Avoidance of donor and donation images

Sixty-two patients reported actively avoiding thoughts about the donor pre-HTX (19.9%, 96 missing responses), with avoidance predominantly occurring directly prior to the HTX (10.6%, *n* = 33; “days before”: 1.3%, *n* = 4; “weeks before”: 1.9%, *n* = 6; “months before”: 3.9%, *n* = 12; “years before”: 2.3%, *n* = 7).

Post-HTX, only slightly more patients avoided such thoughts: 22.1% (*n* = 88, 8 missing responses). In this case, avoidance seemed more likely to occur immediately post-HTX (5.5%, *n* = 22; “days after”: 4.3%, *n* = 17; “weeks after”: 5.5%, *n* = 22, “months after”: 4.8%, *n* = 19; “years after”: 2.0%, *n* = 8).

Avoidance of pre-HTX resulted in the suppression of thoughts in slightly half of patients (*n* = 54, 52.9% vs. *n* = 48, 47.1%), and the same was true for avoidance post-HTX (*n* = 61, 51.3% vs. *n* = 58, 48.7%).

### Feelings experienced in the context of heart transplantation

Table 2 presents the presence of various emotions at the prospect of HTX, following HTX, and the anticipated emotions when thinking about the donor. The predominant emotions before and after the HTX were hope, joy, and gratitude. The most frequently reported aversive emotion was anxiety. When thinking about the donor, gratitude predominated, followed by joy.

### Personal beliefs and perception

Table 3 provides a summary of participants’ personal beliefs and perceptions, revealing a considerable degree of heterogeneity. On the one hand, over 50% stated that the soul does not exist, yet a similarly high proportion considered the heart to be the seat of the soul. Approximately half of the respondents agreed that the soul can influence the body (and vice versa).

### Predictors for donor and donation images

Regarding the pre-HTX time point, gender (female) emerged as the only sociodemographic predictor, and psychological burden appeared to be interrelated with pre-HTX DDI (see Table 4). Thus, the second hypothesis, indicating an association between psychological distress and DDI, can be confirmed. Emotions seemed to play a significant role primarily in thoughts regarding the upcoming surgery, with both positive (e.g., hope) and aversive emotions (e.g., guilt) being of relevance. Anxiety and grief were emotions that predicted pre-HTX DDI at all time points. Additionally, the emotional charge of the heart as the center of emotions and the soul predicted the occurrence of pre-HTX DDI.

Both psychological burden and physical burden led to post-HTX DDI (see Table 4). Consequently, the hypothesis asserting an association between psychological distress and DDI can be confirmed for post-HTX DDI as well. Anxiety and grief continued to be predictors of post-HTX DDI. However, for post-HTX DDI, the emotional charge of the heart as the center of emotions and the soul no longer played a predictive role.

No other variables yielded significant results (see Supplementary Table S1).

## Discussion

### General findings

This study is the first quantitative examination systematically addressing the prevalence and predictors of DDI in a large cohort of *N* = 407 HTX recipients. Most participants reported DDI both before and after the HTX, with a higher frequency observed after the procedure, consistent with findings from previous literature^12^. The prevalence of DDI at 91.0% was notably higher than reported in previous studies^12^. However, it’s worth noting that previous studies were predominantly qualitative, which may account for the variation in estimates. A recent quantitative study in patients after kidney transplantation also demonstrated a similarly high prevalence of DDI^19^. Consequently, DDI may have been underestimated to date.

DDI occurred predominantly on a “daily” basis before surgery, and mostly “less frequently than once a month” after surgery. Moreover, DDI manifested intermittently, occurring immediately before or after, and often in close temporal proximity to HTX. Typically, DDI lasted for “several minutes”. Approximately 20% of recipients reported actively avoiding DDI, suggesting that DDI may act as stressors following the HTX. On the other hand, the decrease of DDI over the years can also be interpreted as an indicator of successful organ integration.

Given the high prevalence of anxiety and emotional preoccupation prior to HTX observed in our sample, the pre-transplant period appears to be a relevant window for psychological intervention. This is in line with current clinical evidence demonstrating that depression and anxiety disorders are common in transplant patients and can negatively affect post-transplant survival^10,26^; depression, for instance, has been consistently linked to increased mortality post-HTX^27^. These findings highlight the clinical relevance of systematic mental health screening and targeted support during the time leading up to transplantation, particularly in patients who report donor-related distress or anxiety.

Psychological distress due to the inpatient hospital stay predicted the occurrence of DDI both before and after the HTX. Additionally, emotions experienced before HTX were associated with higher odds of reporting DDI prior to the HTX, suggesting pre-HTX DDI as a possible side phenomenon within an overall heightened emotional engagement. Anxiety, grief, and fear experienced before HTX, and anxiety and gratitude after HTX, predicted post-HTX DDI. Considering that these emotions, especially anxiety, are typically linked to high levels of uncertainty and low personal control^28^, one could speculate that engaging in DDI may be part of coping with a life-threatening and uncontrollable situation, the grief associated with the loss of one’s organ^29,30^ or the stress related to the presence of positive, aversive, and ambivalent emotions, as previously observed in HTX patients^12,17,29,31^. It can be assumed that this is mainly due to fear such as fear of death, fear of rejection, and fear of complications as pragmatic fears^2,3,12^. Whether (or not) engaging in DDI may alleviate such stress still needs to be further explored.

Interestingly, less than 5% of respondents reported feeling guilty when thinking about the donor, and guilt did not emerge as a significant predictor of DDI. This contrasts with previous reports suggesting that DDI may arise from the idea that someone else has had to die^17,30^. It’s crucial to note, however, that legal regulations in Germany ensure strict anonymity of the donor and cause of death, which could explain the low prevalence and minor role of guilt among the present sample. Notably, guilt is also one of the core dimensions of the TxEQ^23^, which has been used to assess emotional reactions in transplant recipients. The low expression of guilt in our study may indicate that this TxEQ dimension plays a less central role in HTX under the conditions of anonymous donation. However, it also underscores the relevance of exploring DDI as they may capture more nuanced, symbolic, or identity-related emotional processes not fully addressed by existing instruments.

Despite being rare, feelings of grief did predict the occurrence of DDI. Occasional reports in the literature indicate that the loss of one’s organ in favor of a transplanted one, especially when it comes to the heart, can also cause feelings of grief^29,30^.

Other identified predictors of pre-HTX DDI included essentialist beliefs that the heart is the center of emotions and the seat of the soul. This observation aligns with previous reports on the emotionalization of the heart^11,13,32^. Surprisingly, however, such beliefs about the heart played a minor role in the occurrence of post-HTX DDI, and views that the soul has no particular localization predicted the occurrence of post-HTX DDI instead. This could suggest that DDI serve different psychological functions in anticipation compared to after HTX, a phenomenon that future studies should elaborate on.

### Limitations

Since the study was cross-sectional, one can only speculate about the causality of the observed associations. It remains unclear to what extent DDI influence HTX outcomes and whether DDI incur relief (coping) or burden. Future studies should investigate the effects of DDI and their impact of DDI on transplantation outcomes. Another limitation is the use of a self-developed questionnaire. However, it’s important to note that there is currently no instrument available to assess DDI. Consequently, the development of a standardized questionnaire to assess DDI is essential, and this study couldn’t utilize a standardized questionnaire for this reason.

The time since the HTX varied considerably in our cohort and some respondents referring to events that occurred many years ago (e.g., HTX was ≥ 20 years ago in 19% of cases). Consequently, it cannot be excluded that recall biases have influenced the data. Furthermore, as the survey was administered in a fixed paper-and-pencil format without randomization of question order, we cannot fully rule out fatigue effects and systematic dropout toward the end of the questionnaire. We also cannot rule out emotional discomfort -particularly in later sections and more sensitive domains- as potential contributors to the observed response patterns.

Another limitation is that recruitment only took place in one large transplant center, potentially limiting the generalizability of the findings. It also cannot be ruled out that those particularly burdened by the HTX or the DDI were unwilling or unable to participate; thus, further replication of our findings may be needed to rule out selection bias. Future studies should also assess whether patients were on the waiting list vs. high-urgency list, and how long the inpatient stay was before HTX to collect more in-depth burden data.

### Conclusion

This study is the first to quantitatively examine DDI in a large sample of HTX recipients (*N* = 407) – to our knowledge, a systematic study of this magnitude has not been conducted on DDI before. The high prevalence and emotional relevance of DDI highlight their clinical importance. These findings contribute to a deeper understanding of the DDI phenomenon and underscore the need for clinical awareness and further research to improve post-transplant patient care.

**Table 1:** Patients’ characteristics and information about the heart transplantation.

Sociodemographic Variables	*N* (%)
Age *(missing responses)*	*(1)*
Mean (*SD*)	57.91 (14.01)
Median (range)	59 (18–90)
Sex/Gender *(missing responses)*	*(1)*
Male, *n* (%)	294 (72.4)
Female, *n* (%)	111 (27.3)
Diverse, *n* (%)	1 (0.2)
Body Mass Index, mean (*SD*)	25.87 (4.48)
Romantic orientation *(missing responses)*	*(7)*
To opposite gender, *n* (%)	387 (96.8)
To same gender, *n* (%)	9 (2.3)
To males and females, *n* (%)	3 (0.8)
To other genders, *n* (%)	1 (0.3)
German language skills *(missing responses)*	*(2)*
Mother tongue, *n* (%)	357 (88.17)
Fluent, *n* (%)	37 (9.1)
Basic skills, *n* (%)	11 (2.7)
Migration (own person or one of the parents) *(missing responses)*	*(3)*
No, *n* (%)	350 (86.6)
Yes, *n* (%)	54 (13.4)
School years *(missing responses)*	*(9)*
< 12 years, *n* (%)	224 (56.3)
≥ 12 years, *n* (%)	174 (43.7)
Marital status *(missing responses)*	*(1)*
Married/marriage-like, *n* (%)	274 (67.5)
Single, *n* (%)	77 (19.0)
Divorced/widowed, *n* (%)	55 (13.5)
Partnership status *(missing responses)*	*(17)*
With partner, *n* (%)	304 (77.9)
Without partner, *n* (%)	86 (22.1)
Household size *(missing responses)*	*(4)*
1 person, *n* (%)	74 (18.5)
2 persons, *n* (%)	233 (57.8)
> 2 persons, *n* (%)	96 (23.8)
Transplant-Related Variables	
Age at heart transplantation *(missing responses)*	*(6)*
Mean (*SD*)	48.17 (14.24)
Median (range)	52 (1–71)
Waiting time in months *(missing responses)*	*(12)*
Mean (*SD*)	16.33 (27.1)
Median (range)	5 (0.03–180)
Inpatient stay after heart transplantation in days *(missing responses)*	*(16)*
Mean *(SD)*	50.48 (70.7)
Median (range)	35 (4.0-1212.9)
Complications after heart transplantation *(missing responses)*	(30)
No, *n* (%)	167 (44.3)
Yes, *n* (%)	210 (55.7)
Time since heart transplantation until 01/2023 in months *(missing responses)*	*(3)*
Mean *(SD)*	126.01 (100.58)
Median (range)	101.5 (1-399)

**Table 2:** Feelings experienced before/after heart transplantation and anticipated feelings when thinking about the donor.

Feeling *(missing responses)*	Answer Scale			
	Strongly agree *n* ( %)	Agree *n* ( %)	Disagree *n* ( %)	Strongly disagree *n* ( %)
*Feelings at the prospect of heart transplantation*				
Hope *(32)*	282 (75.2)	73 (19.5)	7 (1.9)	13 (3.5)
Joy *(42)*	203 (56.1)	99 (27.3)	33 (9.1)	27 (7.5)
Anxiety *(42)*	94 (25.8)	97 (26.6)	93 (25.5)	81 (22.2)
Shame *(57)*	11 (3.1)	13 (3.7)	61 (17.4)	265 (75.7)
Guilt *(52)*	12 (3.4)	21 (5.9)	56 (51.8)	266 (74.9)
Grief *(50)*	21 (5.9)	68 (19.0)	67 (18.8)	201 (56.3)
Anger *(49)*	6 (1.7)	15 (4.2)	42 (11.7)	295 (82.4)
Fury *(48)*	6 (1.7)	9 (2.5)	33 (9.2)	311 (86.6)
Fear *(49)*	54 (15.1)	75 (20.9)	68 (19.0)	161 (45.0)
Disgust *(49)*	1 (0.3)	2 (0.6)	18 (5.0)	338 (94.2)
Gratitude *(35)*	290 (78.0)	55 (14.8)	11 (3.0)	16 (4.3)
*Feelings after heart transplantation*				
Hope *(24)*	287 (74.9)	78 (20.4)	8 (2.1)	10 (2.6)
Joy *(25)*	284 (74.3)	78 (20.4)	11 (2.9)	9 (2.4)
Anxiety *(35)*	71 (19.1)	77 (20.7)	89 (23.9)	135 (36.3)
Shame *(45)*	9 (2.5)	7 (1.9)	39 (10.8)	307 (84.8)
Guilt *(38)*	13 (3.5)	18 (4.9)	42 (11.4)	296 (80.2)
Grief *(40)*	21 (5.7)	55 (15.0)	65 (17.7)	226 (61.6)
Anger *(49)*	5 (1.4)	10 (2.7)	30 (8.2)	320 (87.7)
Fury *(48)*	4 (1.1)	10 (2.7)	26 (7.1)	328 (89.1)
Fear *(49)*	26 (7.0)	57 (15.4)	61 (16.5)	225 (61.0)
Disgust *(49)*	2 (0.5)	1 (0.3)	13 (3.6)	348 (95.6)
Gratitude *(35)*	328 (85.2)	44 (11.4)	5 (1.3)	8 (2.1)
*Anticipated feelings when thinking about the donor*				
Hope *(81)*	92 (28.2)	95 (29.1)	27 (8.3)	112 (34.3)
Joy *(78)*	113 (34.3)	84 (25.5)	40 (12.2)	92 (28.0)
Anxiety *(80)*	7 (2.1)	15 (4.6)	37 (11.3)	268 (82.0)
Shame *(79)*	5 (1.5)	10 (3.0)	32 (9.8)	281 (85.7)
Guilt *(78)*	10 (3.0)	17 (5.2)	26 (7.9)	276 (83.9)
Grief *(71)*	54 (16.1)	103 (30.7)	33 (9.8)	146 (43.5)
Anger *(76)*	3 (0.9)	3 (0.9)	20 (6.0)	305 (92.1)
Fury *(74)*	1 (0.3)	3 (0.9)	20 (6.0)	309 (92.8)
Fear *(73)*	6 (1.8)	9 (2.7)	26 (7.8)	293 (87.7)
Disgust *(77)*	1 (0.3)	2 (0.6)	11 (3.3)	316 (95.8)
Gratitude *(31)*	323 (85.9)	35 (9.3)	0 (0)	18 (4.8)

**Table 3:** Personal beliefs and Perceptions.

Statements *(missing responses)*	Answer Scale			
	Strongly agree *n* ( %)	Agree *n* ( %)	Disagree *n* ( %)	Strongly disagree *n* ( %)
When a heart has been transplanted, it is indistinguishable from a healthy original. *(30)*	103 (27.3)	107 (28.4)	95 (25.2)	72 (19.1)
With the heart it is like the engine of a car: sometimes parts have to be replaced to keep the car running, but the car remains the same. *(20)*	168 (43.4)	136 (35.1)	44 (11.4)	39 (10.1)
People also feel with their hearts and not just with their heads. *(23)*	143 (37.2)	106 (27.6)	59 (15.4)	76 (19.8)
If a healthy heart could be cloned and transplanted exactly, it would still not be identical to the original. *(60)*	98 (28.2)	82 (23.6)	74 (21.3)	93 (26.8)
The heart is the center of human emotions. *(32)*	77 (20.5)	93 (24.8)	92 (24.5)	113 (30.1)
Body and soul are clearly separated from each other. *(42)*	98 (26.8)	73 (20.0)	86 (23.6)	108 (29.6)
Body and soul are interrelated. *(45)*	138 (38.1)	99 (27.3)	53 (14.6)	72 (19.9)
The seat of the soul is the heart. *(74)*	53 (15.9)	48 (14.4)	67 (20.1)	165 (49.5)
The seat of the soul is the brain. *(79)*	85 (25.9)	77 (23.5)	67 (20.4)	99 (30.2)
The soul has no specific localization, but it exists. *(63)*	139 (40.4)	114 (33.1)	32 (9.3)	59 (17.2)
The soul as such does not exist. *(66)*	32 (9.4)	28 (8.2)	63 (18.5)	218 (63.9)
The soul can influence the body and vice versa. *(49)*	181 (50.6)	122 (34.1)	27 (7.5)	28 (7.8)
The soul can be equated with the personality of a person. *(63)*	108 (31.4)	137 (39.8)	51 (14.8)	48 (14.0)

**Table 4:** Predictors for donor and donation images before/after heart transplantation.

Predictor for donor and donation images	Regression coefficient B (log-odds)	Standard error (SE)	Significance (*p*)
Before heart transplantation			
*Sociodemographic variables*			
Female gender	0.55	0.26	0.033
Psychological burden of inpatient stay	0.09	0.04	0.018
*Feeling at thoughts of the upcoming heart transplantation*			
Hope	1.76	0.58	0.002
Joy	0.63	0.30	0.039
Anxiety	0.78	0.24	< 0.001
Guilt	1.52	0.56	0.006
Grief	1.38	0.33	< 0.001
Fury	2.14	1.05	0.041
Fear	0.50	0.25	0.042
Gratitude	1.64	0.49	< 0.001
*Feelings after heart transplantation*			
Anxiety	0.69	0.24	0.005
Grief	1.01	0.32	0.002
*Anticipated feeling when thinking about the donor*			
Anxiety	1.47	0.64	0.021
Grief	1.20	0.26	< 0.001
*Personal beliefs and perception*			
The heart is the center of human emotions.	0.67	0.24	0.005
The seat of the soul is the heart.	0.83	0.29	0.004
After heart transplantation			
*Sociodemographic variables*			
Psychological burden of inpatient stay	0.20	0.06	0.001
Physical burden of inpatient stay	0.13	0.06	0.033
*Feeling at thoughts of the upcoming heart transplantation*			
Anxiety	1.24	0.43	0.004
Grief	1.64	0.74	0.027
Fear	1.07	0.51	0.034
*Feeling after heart transplantation*			
Anxiety	1.09	0.47	0.020
Gratitude	1.76	0.63	0.005
*Anticipated feeling when thinking about the donor*			
Hope	1.00	0.45	0.027
Grief	2.31	0.75	0.002
Gratitude	1.80	0.57	0.002
*Personal beliefs and perception*			
The soul has no specific localization, but it exists.	0.95	0.40	0.018

The dependent variable is the presence of donor and donation images before/after transplantation “yes” (= 2)/"no” (= 1).

## Electronic supplementary material

Below is the link to the electronic supplementary material.

Supplementary Material 1

## Data Availability

Data sets are available upon request from the corresponding author.
