# Supplementary material for: A large cross-sectional study on the prevalence and predictors of donor and donation images in patients after heart transplantation
Source: Sci Rep. 2025 Jul 1;15:21174. doi: 10.1038/s41598-025-07317-7 (PMC12219023; doi:10.1038/s41598-025-07317-7)
Supplement: Supplementary file 1 — Supplementary Material 1 [file 41598_2025_7317_MOESM1_ESM.pdf]

Prevalence and predictors of donor and donation images (DDI) in patients after heart transplantation – a large cross-sectional study

Supplementary information files

Supplementary Table S1: Non-Significant Results of Binary Logistic Regressions for Donor and Donation Images

**Variable**	**Regression coefficient *B* (log-odds)**	**Standard error *SE***	**Significance *p***
Donor and Donation Images before heart transplantation			
Sociodemographic variables			
Age	< 0.01	0.01	.768
Age at heart transplantation	< 0.01	0.01	.798
Romantic orientation	0.14	0.55	.802
German language skills	0.10	0.34	.777
Migration (own person or one of the parents)	0.26	0.34	.449
School years	-0.23	0.23	.312
Marital status	0.13	0.19	.512
Partnership status	-0.33	0.28	.238
Household size	-0.10	0.17	.550
Weight	< -0.01	< 0.01	.664
Height	< -0.01	< 0.01	.570
Body Mass Index	0.02	0.03	.507
Time since heart transplantation until 01/2023 in months	< 0.01	< 0.01	.930
Waiting time in months	< 0.01	< 0.01	.373
Complications after heart transplantation	-0.06	0.23	.801
Inpatient stay after heart transplantation in days	< -0.01	< 0.01	.349
Physical Burden of inpatient stay	0.07	0.04	.080
Diagnosis of Depression/Anxiety	0.45	0.34	.176
Feeling at thoughts of the upcoming heart transplantation			
Shame	0.86	0.53	.103
Anger	0.90	0.58	.122
Disgust	0.31	1.23	.803
Feelings after heart transplantation			
Hope	0.12	0.52	.814
Joy	0.05	0.48	.910
Shame	0.46	0.55	.405
Guilt	0.48	0.44	.279
Anger	0.34	0.56	.542
Fury	0.23	0.57	.690
Fear	0.46	0.29	.111
Disgust	-1.07	1.23	.386
Gratitude	0.75	0.60	.207
Anticipated feelings when thinking about the donor			
Hope	0.38	0.24	.120
Joy	0.35	.25	.156
Shame	0.25	0.57	.665
Guilt	0.51	0.47	.274
Anger	-0.39	8.3	.639
Fury	0.73	1.16	.529
Fear	0.48	0.70	.493

Disgust	-1.10	1.23	.372
Gratitude	0.59	0.57	.300
Personal beliefs and perception			
When a heart has been transplanted, it is indistinguishable from a healthy original.	0.33	0.23	.148
With the heart it is like the engine of a car: sometimes parts have to be replaced to keep the car running, but the car remains the same.	-0.30	0.29	.304
People also feel with their hearts and not just with their heads.	0.33	0.24	.166
If a healthy heart could be cloned and transplanted exactly, it would still not be identical to the original.	0.35	0.24	.144
Body and soul are clearly separated from each other.	-0.05	0.23	.833
Body and soul are interrelated.	0.13	0.25	.593
The seat of the soul is the brain.	0.09	0.25	.705
The soul has no specific localization, but it exists.	-0.39	0.27	.155
The soul as such does not exist.	0.04	0.32	.897
The soul can influence the body and vice versa.	-0.31	0.33	.736
The soul can be equated with the personality of a person.	0.39	0.26	.135
Donor and Donation Images after heart transplantation (DDI post HTX)			
Sociodemographic variables			
Sex/Gender	0.67	0.46	.144
Age	0.02	0.01	.165
Age at heart transplantation	0.02	0.01	.056
Romantic orientation	17.93	8710.60	.998
German language skills	0.05	0.53	.929
Migration (own person or one of the parents)	-0.11	0.51	.827
School years	-0.04	0.35	.902
Marital status	0.68	0.31	.101
Partnership status	0.68	0.38	.075
Household size	-0.30	0.28	.279
Weight	<0.01	0.01	.629
Height	<0.01	0.01	.674
Body Mass Index	0.02	0.04	.682
Time since heart transplantation until 01/2023 in months	<0.01	<0.01	.089
Waiting time in months	<0.01	0.01	.552
Complications after heart transplantation	-0.07	0.38	.853
Inpatient stay after heart transplantation in days	<0.01	<0.01	.948
Diagnosis of Depression/Anxiety	1.14	0.74	.126
Feeling at thoughts of the upcoming heart transplantation			
Hope	0.67	0.66	.305
Joy	0.48	0.46	.294
Shame	0.78	1.04	.457
Guilt	18.94	7105.18	.998
Anger	-1.34	0.77	.859
Fury	0.27	1.05	.800
Disgust	18.83	23205.42	.999
Gratitude	0.78	0.58	.176
Feelings after heart transplantation			
Hope	-0.47	1.05	.654
Joy	0.73	0.66	.265
Shame	18.86	10377.78	.999
Guilt	18.88	7338.20	.998
Grief	1.37	0.74	.066
Anger	18.81	10377.78	.999
Fury	18.84	110742.02	.999
Fear	1.02	0.66	.101
Disgust	18.81	23205.42	.999
Anticipated feeling when thinking about the donor			
Joy	0.69	0.42	.098
Anxiety	18.71	8569.17	.998
Shame	18.68	10377.78	.999

Guilt	18.72	7735.14	.998
Anger	18.64	16408.70	.999
Fury	18.63	20096.47	.999
Fear	18.66	10742.02	.999
Disgust	18.59	23205.42	.999
Personal beliefs and perception			
When a heart has been transplanted, it is indistinguishable from a healthy original.	0.03	0.38	.936
With the heart it is like the engine of a car: sometimes parts have to be replaced to keep the car running, but the car remains the same.	-0.02	0.45	.957
People also feel with their hearts and not just with their heads.	0.44	0.38	.250
If a healthy heart could be cloned and transplanted exactly, it would still not be identical to the original.	0.44	0.38	.247
The heart is the center of human emotions.	0.49	0.39	.210
Body and soul are clearly separated from each other.	-0.14	0.38	.714
Body and soul are interrelated.	0.68	0.40	.088
The seat of the soul is the heart.	0.19	0.46	.714
The seat of the soul is the brain.	-0.21	0.41	.612
The soul as such does not exist.	-0.82	0.45	.070
The soul can influence the body and vice versa.	0.57	0.46	.226
The soul can be equated with the personality of a person.	0.10	0.42	.821
